# Contact geometry and mechanics predict friction forces during tactile surface exploration

**DOI:** 10.1038/s41598-018-23150-7

**Published:** 2018-03-20

**Authors:** Marco Janko, Michael Wiertlewski, Yon Visell

**Affiliations:** 1 Drexel University, Department of Electrical and Computer Engineering, Philadelphia, 19104 USA; 2 Aix Marseille University, CNRS, ISM, Marseille, France; 3 University of California, Department of Electrical and Computer Engineering, Media Arts & Technology Program, and Department of Mechanical Engineering, Santa Barbara, California 93106 USA

## Abstract

When we touch an object, complex frictional forces are produced, aiding us in perceiving surface features that help to identify the object at hand, and also facilitating grasping and manipulation. However, even during controlled tactile exploration, sliding friction forces fluctuate greatly, and it is unclear how they relate to the surface topography or mechanics of contact with the finger. We investigated the sliding contact between the finger and different relief surfaces, using high-speed video and force measurements. Informed by these experiments, we developed a friction force model that accounts for surface shape and contact mechanical effects, and is able to predict sliding friction forces for different surfaces and exploration speeds. We also observed that local regions of disconnection between the finger and surface develop near high relief features, due to the stiffness of the finger tissues. Every tested surface had regions that were never contacted by the finger; we refer to these as “tactile blind spots”. The results elucidate friction force production during tactile exploration, may aid efforts to connect sensory and motor function of the hand to properties of touched objects, and provide crucial knowledge to inform the rendering of realistic experiences of touch contact in virtual reality.

## Introduction

The tools we touch, grasp and manipulate often have topographical features such as ridges, bumps and texture that are meant to guide our gestures and provide crucial information on their nature and function. As an example, the rubbery curve on a quality pen provides grip and indicates where to position our fingers for optimal manipulation. These features are often recognized by touch alone from the deformation imposed by the skin in contact with the feature. While static shape perception can be attributed to the indentation (i.e. normal deformation) of the skin, the dynamic perception of these features is due to the complex interactions that result from sliding, in particular to the modification of frictional forces. Complex frictional forces are involved in both static and sliding contact between the finger pad and a touched surface. Friction plays an essential role in object grasping and manipulation, interrelating the grip and load forces that are exerted by the fingers and that must be coordinated in order to prevent slipping^1^.

Surface curvature, roughness, bumps, and ridges can enhance friction forces, aiding grasping and fine manipulation^2,3^. Sensing these features is facilitated by dynamic tactile signals from thousands of mechanoreceptive afferents that innervate the finger pads^4^, allowing the brain to identify surface features and select an appropriate grasp. Friction forces are crucial for such acts of discriminatory touch. Sliding our fingers over the surface of an object, we obtain perceptual information about roughness^5^, shape^6^, adhesion^7^, and texture^8^. Local surface features, such as edges, are also reliably encoded in population responses of tactile afferents in the fingertip^9^. Variations in friction forces yield high-frequency, low-amplitude skin deformations that propagate throughout the hand^10^, exciting mechanoreceptive afferents within and beyond the finger pad, and further facilitating surface texture discrimination^8,11^.

While the relevance of friction forces to sensory and motor functions of the hand is well established, our understanding of how time-varying friction forces are produced during contact with non-flat surfaces is incomplete. This can partly be attributed to the complex dynamics governing the tribology of the finger pad^12^, including the moisture content of the fingertip skin, which can affect the mechanics and dynamics of frictional sliding sliding^12–14^, static to sliding transitions^15–17^, and the presence of non-Coulombic behavior^18^. However, effects due to variations in surface geometry are also very difficult to account for. Even very controlled sliding of a finger on non-flat surfaces, such as sinusoidal gratings^19–21^ or braille dots^22^, yields forces that fluctuate in time and that vary greatly between trials. Existing mechanical models are unable to anticipate these forces from surface properties and interaction parameters, such as finger velocities and applied forces, except for surfaces whose heights vary slowly relative to the size of a finger^23^.

It is unclear whether these force fluctuations are attributable to finger pad dynamics, contact mechanics, or to other tribological effects, but one indication of what is occurring may be the development of complex patterns of contact between the finger and the surface^15,17,24^, which arise due to the limited elasticity of fingertip tissue. The resulting multi-contact interfaces vary with position and pressure^25^, and can evolve rapidly in time. The significance of these multi-contacts has also been noted in perception studies, where it was observed that they could account for the dominant effect of high relief features on roughness perception during tactile exploration of rectangular gratings^24^. However, the evolving regions of contact are difficult to capture during tactile exploration, and there has been little prior empirical investigation of the role of contact geometry in shaping forces felt by the finger.

This problem of predicting how dynamic friction forces reflect the topography of touched surfaces is also highly relevant to the design of human interactive technologies, especially for the expanding category of surface haptic displays – touch screen displays and interfaces whose friction coefficients can be electronically controlled in order to provide tactile feedback via ultrasound^26–28^ or electrostatic adhesion^29–31^. Existing simulation methods are based on reproducing either pre-recorded or synthetically generated friction forces^32–34^, yielding feedback that is either rigidly specified or unphysical and artificial, unlike those that would be produced during sliding on any real surface. Thus, here too, there is a need for a greater understanding of friction forces produced during tactile exploration of non-flat surfaces.

Several challenges are encountered in modeling the mechanics of finger-surface interactions, including the complex dynamics of finger pad friction^12^, the nonlinear stiffness and viscoelastic characteristics of the tissues^35,36^. Analytical modeling of the fingertip has yielded useful insight for quasi-static or dynamic indentation problems^35–38^, but the results have limited utility for understanding dynamic deformation of finger tissues induced during frictional sliding. Of the many studies of frictional properties of the primate fingerpad, few have addressed sliding contact on realistic surfaces. Volumetric numerical simulations of fingertip sliding on different surfaces can predict a restricted range of observed phenomena^39–41^, such as the development of nonlinear oscillations, but fail to capture the variability of sliding on real surfaces^42^, and are strongly simulation dependent – i.e., they cannot predict force production from surfaces that have not been simulated. Local surface descriptors, such as contact orientation of the surface at a contact point^6,43,44^, have been proposed in order to account for the effect of surface shape on force production, but have not been applied to distributed contact with the finger pad, where the slope may vary within a single contact region, and may not be defined at locations where contact is broken.

In order to account for friction force production between the finger pad and non-flat surfaces, we measured forces and contact interfaces during frictional sliding on relief surfaces, using synchronized force measurements and a fronto-parallel high-speed video capture configuration. Using image processing methods, we tracked the geometry of contact interfaces from the latter, and used the resulting data to estimate parameters of a spatially distributed frictional model. As we show, this model, which accounts for surface shape and contact mechanical effects, could accurately predict forces that were felt during sliding on a variety of surfaces at different speeds.

## Results

**Figure 1:**
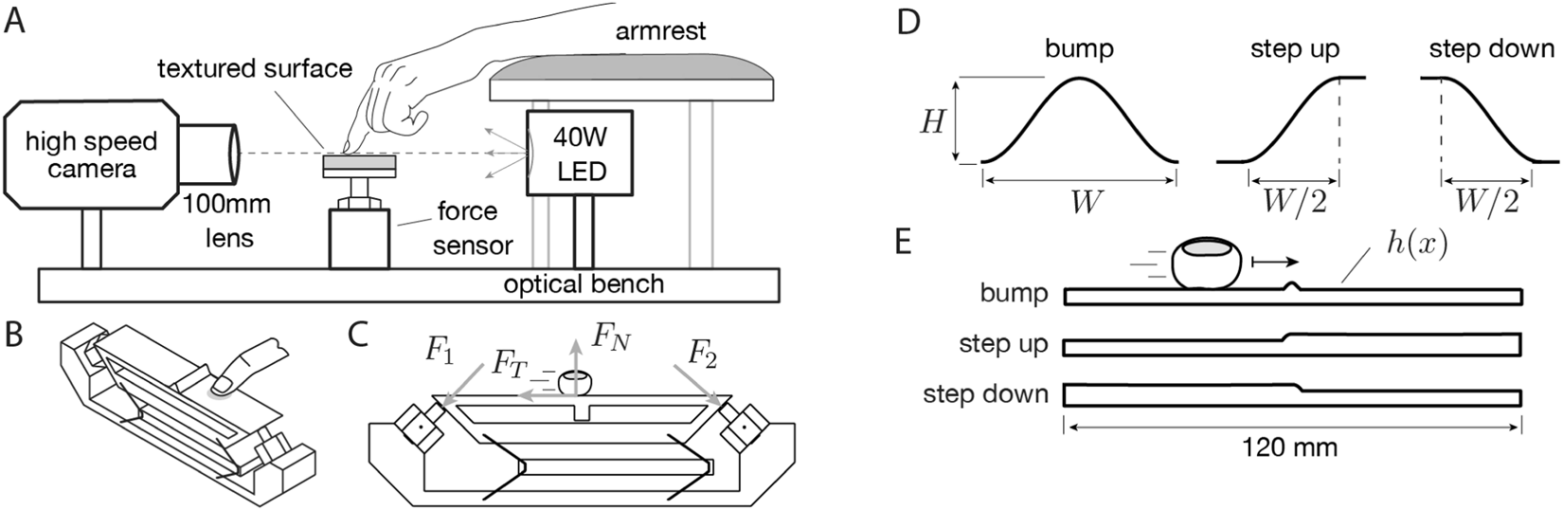
Experimental apparatus and relief surfaces. (**A**) Measurement system, side view. A high speed camera, two-axis force sensor, LED light source, and tactile surface were mounted on an optical bench, and aligned ensuring accurate fronto-parallel imaging of the finger contour. (**B**) Isometric view of the two-axis force sensor. (**C**) Frontal view of the force measurement device with force decomposition. (**D**) Surface center feature geometric specification. (**E**) Front view illustrating the surfaces; These shapes are replicated for two widths (*W* = 2 mm, 4 mm) and heights (*H* = 0.2 *W*), yielding six surfaces in total.

We captured forces, finger location and deformation during sliding contact of the index finger of two individuals on relief surfaces at three different speeds (40, 80, or 120 mm/s), using force sensing, high speed video capture (Fig. 1A–E), and video analysis (see Methods). We used image analysis to track the center of the finger, *x*(*t*), during sliding (see Methods). The six surfaces were macroscopically flat with localized features in one of three shapes (a bump, a rising step, or a falling step) and either of two scales (width 2 mm or 4 mm).

**Figure 2:**
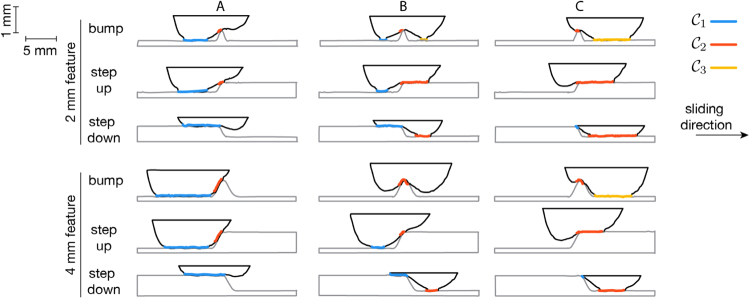
Example patterns of finger-surface contact, extracted via high-speed video capture and analysis (see Methods), at three successive instants (**A**–**C**) for each of the six relief surfaces used in the study, exemplifying three different contact conditions. At initial finger contact (**A**) with the relief feature, the contact interface ${{\mathscr{C}}}_{1}$ of the finger with the flat region to the left of the relief feature is large, and (**B**) decreases as the finger traverses the inclined region, and becomes supported on the relief feature, where a second disconnected contact interface ${{\mathscr{C}}}_{2}$, develops. For the bump surfaces, this is followed by the development of a third disconnected contact region, ${{\mathscr{C}}}_{3}$, which forms as the finger descends a second slanted region.

**Figure 3:**
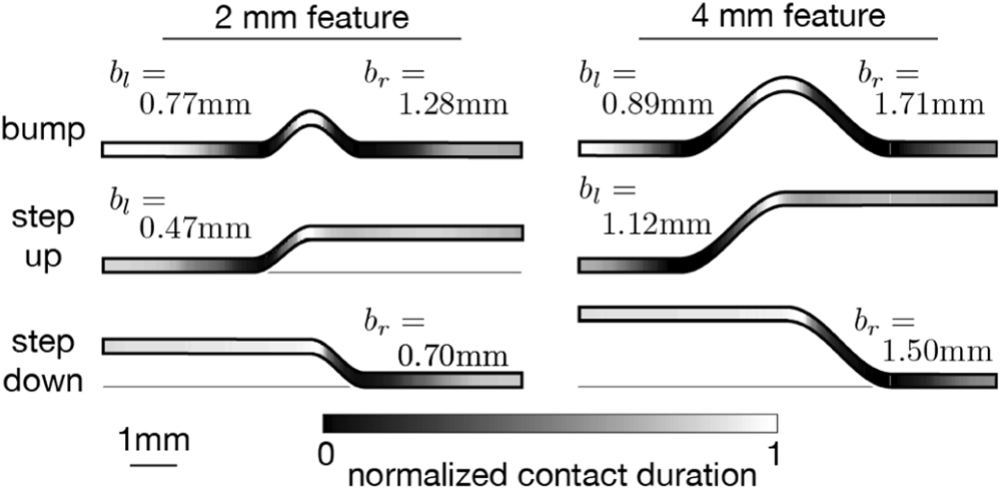
Normalized proportion of time of contact between the finger and surface locations (both subjects, all trials). At the highest level on the scale (1, white), the finger was in contact with the surface for the highest proportion of time, while at the lowest level on the scale (0, black), the surface was never contacted by the finger. In each trial, finite width regions of every surface satisfied this last condition (two regions in the case of bumps); we refer to them as “tactile blind spots”. Their widths ranged from 0.47 mm (Step Up surface, 2 mm scale) to 1.71 mm (Right Bump surface, 4 mm scale).

The contact interface between the finger and surface varied with the time-dependent position of the finger (Fig. 2). Initially, finger-surface contact was restricted to a flat region ${{\mathscr{C}}}_{1}$ to the left of the relief feature. As the finger came into contact with the curved region, additional, disconnected contacts developed, due to finite stiffness of finger tissues, which limit its ability to conform to curved areas of the surface. A second contact, ${{\mathscr{C}}}_{2}$, disconnected from the first, thus developed as the finger ascended (Bump and Step Up surfaces) or descended (Step Down surfaces) the incline. For the bump surfaces, this was followed by the development of a third disconnected contact region, ${{\mathscr{C}}}_{3}$, which formed as the finger descended the raised feature. The disconnections were maintained at all times, for every trial, sliding speed, and subject. Consequently, despite the slow change in height of these surfaces, whose maximum slope angle did not exceed 0.56 radians (32 degrees), there were convex regions of each, nearest to the curved region, that were never contacted by the finger of either participant (shaded black in Fig. 3). We refer these as “tactile blind spots”, because they remained “invisible” to touch contact in all trials. Their widths ranged from 0.47 mm (Step Up surface, 2 mm scale) to 2.6 mm (Bump surface, 4 mm scale). While the smallest bumps created a maximum of three simultaneous contacts, all other surfaces yielded no more than two at any time.

We computed the lengths *L*_*i*_ of each contact region ${{\mathscr{C}}}_{i}$ in each frame, as measured via fronto-parallel video, in order to characterize how they evolved during sliding. The resulting patterns were highly stereotyped for each surface (Fig. S1), varying little between speeds. There were modest differences between subjects (Table S1), likely due to variations in finger shape or stiffness. As the finger traversed the curved section of the surface, the lengths of the first contour (${{\mathscr{C}}}_{1}$) and last (${{\mathscr{C}}}_{2}$ or ${{\mathscr{C}}}_{3}$), on or off of the curved region, increased or decreased in nearly identical fashion on every trial for each surface, remaining restricted to a tightly bounded range (Fig. S1), and further highlighting the relatively invariant relation between surface shape and finger-surface contact evolution.

**Figure 4:**
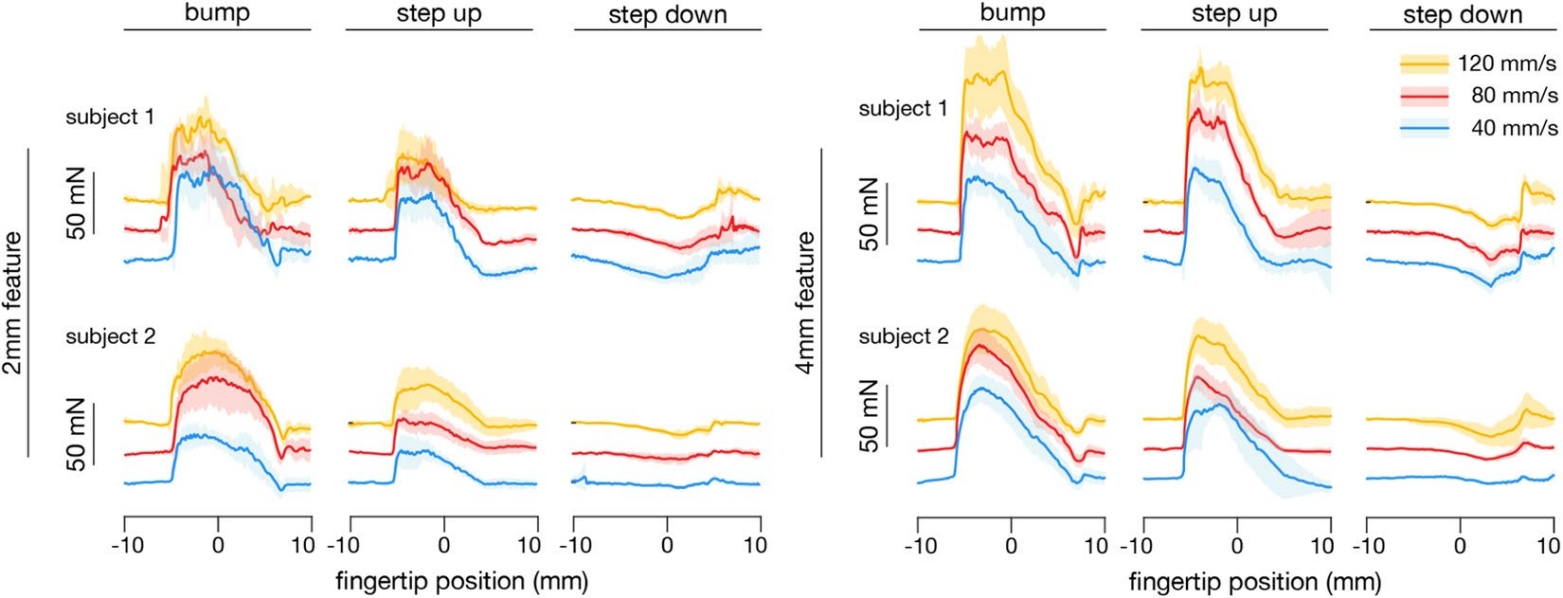
Measured forces *F*_*T*_(*x*) grouped by surface and subject (15 trials per case). Mean of 5 trials at 40 mm/s in red, mean of 5 slides at 80 mm/s in blue and mean of 5 trials at 120 mm/s in orange. Shaded regions: 1 standard deviation. The forces at each speed are offset by 25 mN to aid readability. (For a superimposed force plot, see Supplementary Information, Fig. S6).

Force components *F*_*T*_(*x*) tangent to the surface fluctuated from trial to trial (Fig. 4), but yielded spatial variations in force that varied consistently with the geometry of the touched surface, with some inter-subject differences. Raised features produced sharp increases in force magnitude, the latter rising on the order of 50 mN within about 1 mm, at locations when the finger came in contact with the surface feature in the region of positive surface slope, *dh*/*dx* > 0, where *h*(*x*) is the surface height function. High speed video analysis revealed the increases in force to coincide with the compression of the leading side of the finger as it contacted the ascending slope of the surface (Fig. 2A). There were typically small but consistent transient decreases in force (approximately 5 to 20 mN), followed by increases (5 to 50 mN), as the finger descended incline features, possibly due to a transient increase in normal force, and contact area, of the fingerpad against the recessed flat region (Fig. 2C). Forces were similar at different sliding speeds (40, 80, and 120 mm/s), with slightly higher variability at higher speeds, indicating that hysteresis friction played only a modest role. Consequently, in modeling force production, we omitted any explicit dependence on sliding speed.

### Modeling frictional forces

To clarify the relation between contact geometry and force production during tactile exploration of relief surfaces, we developed a simplified analytical model that could be used for comparison with our measurements. As we demonstrate, this model agrees with the observed spatial variations in friction forces. It accounts for two main features of finger-surface interactions, namely the geometry of contact between the skin and surface, and the development of frictional forces at the boundary between the two. Within the two-term non-interacting model, the frictional force *F* can be assumed to result from the sum of interfacial *F*_*int*_ and deformation *F*_*def*_ components^45,46^

1 $$F={F}_{int}+{F}_{def}$$

**Figure 5:**
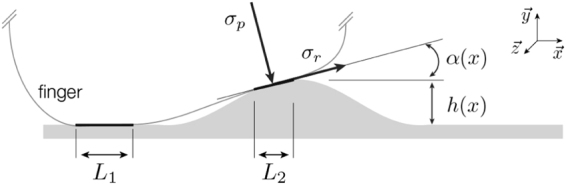
Contact between the finger and surface yields distinct regions of contact (lengths *L*_*i*_), and local surface stresses *σ*_*p*_ and *σ*_*r*_ normal and tangential to the surface at a point within each region. Variations in surface height *h*(*x*) are accompanied by changes in slope *α*(*x*), with *dh*(*x*)/*dx* = tan *α*(*x*).

The interfacial component *F*_*int*_ corresponds to the energy dissipated in the breaking of molecular bonds formed transiently between the sliding surfaces due to short range attractive forces. For rough surface contacts, the applied load per unit area *σ*_*p*_(*x*) acts in the direction normal to the surface at *x*, while the homogenized interfacial stress *σ*_*r*_(*x*) is tangent to the surface in the projection plane; the angle of the surface with the horizontal is *α*(*x*) (see Fig. 5). The normal and tangential stress are related by a Coulomb-Amonton term *σ*_*r*_ = *μσ*_*p*_, with a coefficient of friction *μ* that is independent of the applied load. While this assumption is common, with some theoretical and empirical support^46,47^, it has some limitations (see Discussion). The net interfacial force in the direction of motion is obtained by projecting the local stress components onto the *x*-direction and integrating over the contact area, ${\mathscr{A}}$. This yields

2 $${F}_{int}={\int }_{{\mathscr{A}}}\,{\sigma }_{p}(x)\,\{\sin (\alpha (x))+\mu \,\cos (\alpha (x))\}\,dxdz$$

The deformation component *F*_*def*_ is due to the energy dissipated by subsurface viscoelastic deformation near the leading edge of the contact. As reflected in our measurements (Fig. 4), this is largest when there is interlocking between the contacting bodies, corresponding to a raised surface feature, and yields a local friction force component proportional to the product of the net vertical stress *σ*_*y*_ and the tangent of the angle *α*(*x*) of the surface with the horizontal^25^, which is also the slope, tan *α*(*x*) = *dh*(*x*)/*dx*, where *h*(*x*) is the height function (see Fig. 5). We integrated local deformation stresses across the contact interface, and projected these onto the *x*-direction, since this yielded the best fit to our observations, yielding a force term of the form

3 $${F}_{def}\propto {\int }_{{\mathscr{A}}}\,\frac{dh(x)}{dx}\,\{\sin (\alpha (x))+\mu \,\cos (\alpha (x))\}\,dxdz$$

To account for hyperelastic tissue deformation, we investigated nonlinear dependencies on slope *dh*(*x*)/*dx*, but these did not yield better agreement with our observations (see Fig. S2, Supplementary Information). For simplicity, we modeled normal stress *σ*_*p*_(*x*) as a constant pressure *p*_0_, drawing on an analogy with a fluid-filled membrane in equilibrium that has proven useful in prior fingertip models^36^. Since the relative importance of interfacial and deformation components is unknown, we introduce a constant weight, *p*_1_, for the latter. To match our fronto-parallel measurement configuration, we interpret the area integral as spanning an effective contact length, i.e. as a sum of integrals over ${N}_{{\mathscr{C}}}$ disconnected contact regions ${{\mathscr{C}}}_{i}$ of lengths *L*_*i*_ with effective size *D* in the *z* direction. For simplicity, we assume *D* to be a constant and, without loss of generality, absorb it into the parameters *p*_0_, *p*_1_ introduced above. Putting these together, we obtain

4 $$F={F}_{int}+{F}_{def}=\sum _{i\mathrm{=1}}^{{N}_{{\mathscr{C}}}}\,{\int }_{{{\mathscr{C}}}_{i}}\,({p}_{0}+{p}_{1}\frac{dh(x)}{dx})\,\{\sin (\alpha (x))+\mu \,\cos (\alpha (x))\}\,dx,$$

We sought to evaluate this explanatory mechanical model by comparing it with the observations from our experiments. The height functions *h*(*x*) of our surfaces are known, as are the slope *dh*(*x*)/*dx* and angle *α*(*x*) = atan(*dh*/*dx*) (see Methods). The contact surfaces ${{\mathscr{C}}}_{i}$ are obtained from the video analysis. Because the participants explored the surfaces with their fingers unconstrained, the pressure, represented by *p*_0_, and contact conditions, which involve *p*_1_, could vary from trial to trial. We estimated them from the measurements in each trial (see Methods). We also estimated a constant value of the friction coefficient *μ* for each subject, because we took measures to control friction during the experiment.

**Figure 6:**
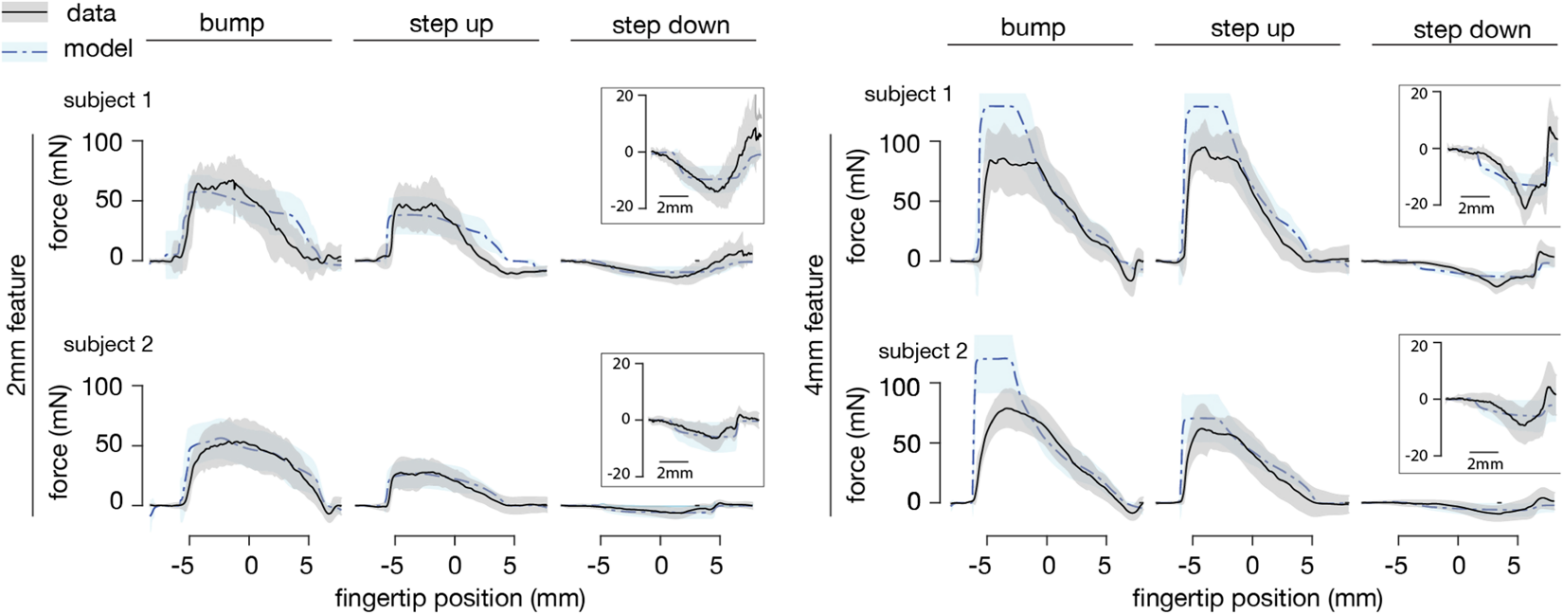
Comparing measured and modeled frictional forces. Measured forces *F*_*T*_(*x*), mean of 15 trials in each condition (black). Model estimates ${\bar{F}}_{T}(x)$, mean of 15 trials in each condition (blue). Shaded regions: 1 standard deviation. Inset provides further detail for the Step Down surfaces, which elicited smaller forces.

This yielded model predictions for friction forces patterns that varied with finger position, and approximately matched the measured forces in all conditions, for both subjects (Fig. 6). The highest force values, produced during the initial contact phase between the finger and surface, were overestimated for the coarsest surfaces, but at other instants, and in all other conditions, the model predictions $\hat{F}(x)$ were within one standard deviation of the measured values *F*(*x*). The modeled forces exhibited fluctuations, due to variations in finger-surface contact, that were similar in scale to the measurements (Fig. 6, shaded regions). A Pearson correlation analysis between modeled and measured force patterns yielded values *ρ* that were greater than 0.75 in all but one subcondition, accounting for all 180 trials (Table 1). Results were similar at all three speeds tested. Correlations were lowest for the Bump surfaces (both spatial scales), reflecting the variable quality of the correspondence between model and measurements (Fig. 6).

**Table 1:** Measured and estimated forces, *F*_*T*_ and ${\hat{F}}_{T}$, were significantly similar in all conditions (Pearson’s correlation value, *ρ*), for both surface heights, both subjects. Data from all three speeds is grouped.

	Subject 1						Subject 2						Both subjects					
	2 mm			4 mm			2 mm			4 mm			2 mm			4 mm		
	Bump	Step	Step	Bump	Step	Step	Bump	Step	Step	Bump	Step	Step	Bump	Step	Step	Bump	Step	Step
		Up	Down		Up	Down		Up	Down		Up	Down		Up	Down		Up	Down
*ρ*	0.74	0.86	0.75	0.83	0.89	0.86	0.88	0.92	0.86	0.65	0.77	0.90	0.79	0.86	0.81	0.75	0.86	0.89
*p* < 0.01	$\ast$	$\ast$	$\ast$	$\ast$	$\ast$	$\ast$	$\ast$	$\ast$	$\ast$	$\ast$	$\ast$	$\ast$	$\ast$	$\ast$	$\ast$	$\ast$	$\ast$	$\ast$

## Discussion

We investigated the frictional sliding of the fingertip against relief surfaces, using high-speed video and force measurements, and analyzed the results with the aid of a friction force model that accounted for interfacial and deformation effects. We observed that the forces vary greatly from one sliding interaction to the next (Fig. 4), with patterns that do not bear an obvious relation to the surface geometry. This is consistent with previous measurements of sliding contact forces between a fingertip and high relief features in sinusoidal gratings^20^ or braille dots^22^. The spatial patterns of the forces that we observed depended on the shape of the surface, and to a lesser degree on the speed of sliding.

By tracking the finger and surface using high speed video, we observed the spatiotemporal pattern of finger-surface contact during sliding. The geometry of contact evolved during the course of motion in a manner that was highly stereotyped between trials (Fig. S1). Multiple regions of contact developed between finger and surface near surface concavities (Fig. 2), due to the stiffness of the finger tissues. Despite the modest curvature of the surfaces, there were regions of each that were never contacted by the finger of either subject (Fig. 3). These regions were comparable in size to the surface relief features themselves. We refer to these as “tactile blind spots”, because they represented surface regions that could not be felt by the finger during sliding, due to the absence of contact. Although such regions do not directly contribute to friction, it can be concluded that they affect the distributions of frictional forces at the fingertip. From a perceptual standpoint, Lederman and Taylor previously remarked upon the relevance of contact gaps for the perception of roughness during the tactile exploration of high relief surface textures^24,48^, although the mechanical explanation for this remained largely unexplored. While subjects may or may not be consciously aware of these contact gaps (and we suspect not), their presence undoubtedly affects both friction and stress distributions in the fingerpad. As such, they may significantly affect the tactile perception of relief surfaces. For example, a gap in contact could increase stress gradients, thereby augmenting the perception of surface features. Future investigations of the saliency of contact gaps to mechanics and perception could help to elucidate these issues. From an applications standpoint, it is also noteworthy that existing surface haptic displays are unable to reproduce such features.

We used the tracked contact interfaces and force measurements to investigate the geometric and mechanical origin of sliding friction forces, by developing a model (Eq. 4) that accounts for distributed finger-surface contact, and contributions of interfacial and deformation contact stresses. While deterministic, the model reproduces force fluctuations due to variations in finger-surface contact, including at multi-contact interfaces. The measurements indicated that even small changes in contact, such as at the leading edge of a raised feature, could elicit large changes in friction forces. Modeled force fluctuations were also similar in scale to the measurements.

Friction forces in the experiment varied with the shape and scale of the surfaces. Initial contact with highest features elicited the largest forces. During contact with surface features, the force variations extended over a length scale of about 10 mm (Fig. 4), considerably larger than that of the surface features (2 mm and 4 mm), due to the finite size of the finger. While the finger-surface contact areas were very consistent from trial to trial, force variations were much larger, with standard deviations reaching 50% of the force magnitude (Fig. 4). Forces produced during sliding on the Step Down surfaces were only about 20% as large in magnitude as the Step Up surfaces, which were geometrically identical, but reversed in spatial orientation. This difference can be attributed to the absence of deformation forces produced via interlocking between the finger and raised features when traversing the Step Down surfaces. This interpretation appears to be consistent with the predictions of the model, which suggested that deformation forces *F*_*def*_ were almost an order of magnitude larger than interfacial forces *F*_*int*_ during contact with raised features (Step Up, Bump), and were negligibly small for the Step Down surfaces (see Fig. S3).

While the model was generally able to capture the observed force patterns (Fig. 6), the largest discrepancies were observed at initial contact with the highest raised features. These large forces could be due to unmodeled effects, such as static to sliding contact transitions, although these cannot be reliably identified using our apparatus. They are not captured in the proposed model, but would be important in a complete account of tactile exploration at low speeds. Among other possible sources of discrepancies of the model predictions with the measurements are mechanical effects of sliding friction that are not accounted for, and uncontrolled aspects of the sliding interactions and stimuli. At low forces, the coefficient of friction *μ* between the finger and a counter surface varies with normal force^18^. For the magnitudes of normal force studied here (approx. 0.3 N), the rate of change of *μ* appears to be slow – less than 20% change if the force is doubled or halved – but could affect the agreement with the measurements, and (more importantly) the applicability of the model to a range of force levels.

In the experiment, the finger was unconstrained, and participants were trained to perform sliding at specified speeds and normal forces. While this ensured that the interactions more accurately reflected those that occur during natural tactile exploration, it also meant that the speed and applied forces varied (Fig. S5), due to differences in motor performance and to forces produced during interactions between the finger and relief features. While this could affect the forces, our measurements did not reveal any consistent effect of sliding speed (Fig. 4). Indeed, during contact with relief features, the modeling results suggest that the forces were dominated by fingertip deformation (Fig. S3). In addition, although we controlled moisture during the experiment, this model does not account for the hydration of the fingertip. Moisture can greatly affect the compliance of the outer layers of the skin, consequently increasing friction forces, and can also introduce additional time-scales to the dynamics^12,18,35^. Under large displacements, the fingerpad exhibits hyperelasticity^40,49^ which is also not accounted for here, nor is the variation of tissue stiffness with finger orientation^50^, or viscoelasticity^50^. The ambiguous effect of sliding speed on these results call into question whether viscoelastic effects contributed significantly, and may be consistent with previously reported results^51,52^. Future investigations may be needed in order to more fully account for such effects. Finally, while these results are based on experiments that involving many measurements (on the order of 300,000 images, for example), the data were collected from only two participants interacting with three surfaces at three speeds. Future studies aimed at assessing these results across conditions and individuals is warranted in order to assess the generalizability of these observations.

The results of this study contribute toward our understanding of sliding friction forces during tactile exploration. They may aid efforts to understand sensory and motor functions of the hand, and to relate them to properties of touched objects. Finally, predictive modeling of contact interaction forces is needed in order to inform the rendering of realistic experiences of touch contact in virtual reality.

## Methods

### Measurement apparatus

The force sensor, a custom two axis load cell, consisted of an aluminum tray resting on two piezoelectric transducers (Model 9712A5, Kistler Instruments, Winterthur, Switzerland; resolution 45 *μ*m) through hemispherical contact buttons. The piezoelectric transducers were fixed to a base at 45° angles with respect to the horizontal plane. The tray was constrained in all but the horizontal and vertical directions through a compliant mechanism of flexures with stiffness negligible compared to that of the piezoelectric transducers, see Fig. 1B,C. Signals from the piezoelectric transducers made it possible to compute normal *F*_*N*_ and tangential force *F*_*T*_ on the tray via simple trigonometry, i.e. ${F}_{N}=({F}_{1}+{F}_{2})/\sqrt{2}$ and ${F}_{T}=({F}_{1}-{F}_{2})/\sqrt{2}$. The tray was 120 mm long and 25 mm wide. The force measurement device was machined from 6010 aluminum alloy using Electrical Discharge Machining (EDM), the flexures were constructed from 0.25 mm type 1095 spring steel. The device had a nearly flat frequency response from 15 to 500 Hz. The piezoelectric transducers were powered using a compliant power supply (Model 5134, Kistler Instruments, Winterthur, Switzerland), and signals from each sensor were conditioned and digitized with a 0.1 ms sample period (10 kHz sample rate) and 16 bits quantization using a data acquisition hardware (NI-6229, National Instruments Inc., Austin, TX). Three zero-phase notch filters centered around 50 Hz, 150 Hz and 300 Hz, were used to remove power supply interference.

Fronto-parallel video of the finger was captured using a high-speed camera (Phantom Miro M110, Vision Research Inc., Wayne, NJ), with 1 ms sample period (1 kHz sample rate) and resolution 1280 × 720 pixels. The camera position centered on the interaction region, yielding an effective spatial resolution of 0.01 mm. Illumination was provided by a 40 W LED light source (No. 5 LED, HS Vision GmbH, Ettlingen, Germany) generating a light beam that covered the entire aperture of the lens of the camera, and yielding high-speed video that consisted of the background (bright light), the contour of the surface profile (dark area) and the shaded area caused by the fingertip obstructing the light. This configuration (Fig. 1A) made it possible to accurately track the fingertip contour and its evolution during the sliding contact.

### Relief surfaces

We fabricated solid relief surfaces by machining rectangular (120 mm × 25 mm) aluminum plates with localized relief features. We modeled the samples parametrically and fabricated them from 6061 aluminum alloy using electrical discharge machining (Model HS-3100, Brother Intl.; Accuracy and roughness approx. 1 *μ*m) yielding a macroscopically artifact-free finish. We included three types of relief features, located at the center of otherwise flat surface samples: Bump, a Step Up edge, and a Step Down edge, see Fig. 1D,E. Two widths for each relief feature were used, 2 mm and 4 mm, yielding a total of 6 surfaces. All shapes had a raised cosine profile *h*(*x*), with heights *H* equal to 20% of their widths *W*.

### Procedure

The experiments were approved by the institutional review boards of both Drexel University and Aix Marseille University, with the informed consent of all participants (experiments 1610004894 and RO-2016/36). All methods were performed in accordance with the relevant guidelines and regulations. We captured video and force data as each of two participants (male, ages 29 and 35) slid their index finger on the surface samples (Fig. 1D,E) five times. Sliding was performed at each of three speeds (40 mm/s, 80 mm/s and 120 mm/s) for a total of 15 trials per surface per person, and 180 trials in total. Sliding speed was assessed after the experiment (Fig. S5). The sliding direction was always the same, from the participants left to right side. The normal force was prescribed to be 0.3 N, a value that is typical of forces observed in prior studies of tactile exploration^53^. Participants were trained to produce this force level prior to the experiment, using a force sensor, and continued until they were able to maintain the force within a range ±50 mN. An audio metronome aided them in maintaining the prescribed average sliding speed in each trial. In the experiment, the finger was otherwise unconstrained (Fig. 1A), in order to ensure that the interactions more accurately reflected those that occur during natural tactile exploration, In order to maintain a constant level of friction, the fingers were cleaned with isopropyl alcohol before each recording trial. The surfaces were treated with a small amount of talc to reduce stick-slip effects.

### Image analysis

**Figure 7:**
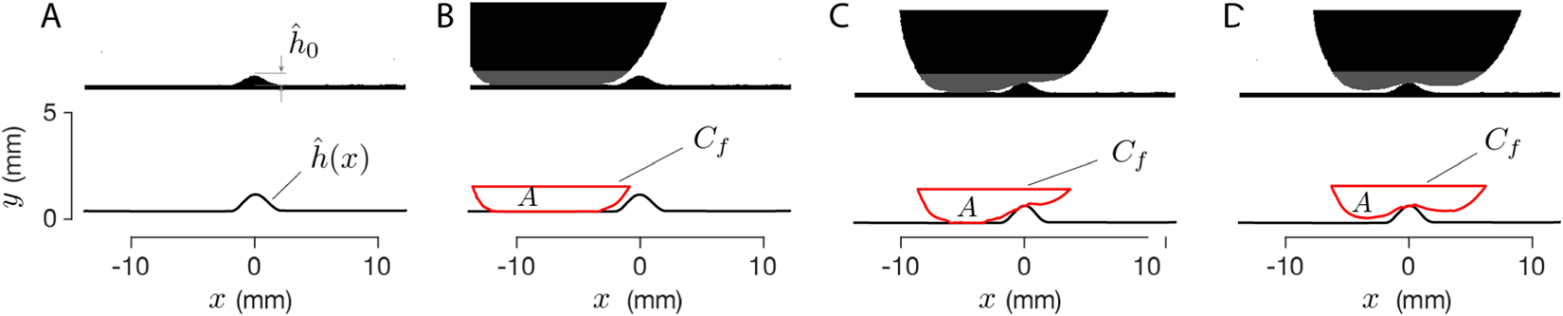
Image analysis procedure (illustrative, 4 mm sinusoidal bump). (**A**,**B**) First frame of the video sequence without (**A**) and with (**B**) finger, used to calibrate the length scale, identify the surface geometry, and initiate the finger contour tracking. (**C**,**D**) Tracked finger contour *C*_*f*_, enclosing area *A*, as the finger contacts the surface feature.

The high speed video data for each trial consisted of a sequence of grayscale images – video frames sampled in time. Each image had a resolution of 1280×720 pixels. They were cropped after recording to the center 1260×360 pixels to remove unwanted background and then were converted to binary images using a threshold operation. Each pixel in the resulting binary images represented either background illumination (1) or shade from the finger and/or surface profile (0). We determined the size of one pixel in the image plane (which was approximately 23 *μ*m) from both the maximum height ${\hat{h}}_{0}$ in pixels, of the observed shape $\hat{h}$ and the known height of the surface *H* expressed in mm, leading to a precise calibration of the spatial dimensions, see Fig. 7A.

We processed the binary images in order to extract outlines of the finger and surface profile, by using the Moore-Neighbor tracing image boundary detection algorithm modified by Jacob’s stopping criteria^54^, implemented in custom software using an image processing library (Matlab, The Mathworks Inc., Natick, MA). We extracted curves that delineated the contours representing the fingertip, surface, and the contact interface between them, and tracked their motion over time.

The initial surface profile contour, a geometric curve $\hat{h}$, was extracted from the first frame of the video, before the finger contacted the surface feature (e.g., Fig. 7B). To aid the tracking of finger contours, we modeled the tissue as approximately incompressible, so that a deformation from interaction with the surface profile caused tissue to be displaced to other regions. We computed the area *A* (Fig. 7B) enclosed by the fingertip contour, and intersected it with a horizontal line located 0.4 mm above the maximum height of the surface feature. For each subsequent frame, the region of interest from the fingertip was obtained as a closed geometric curve *C*_*f*_ resulting from the intersection between the fingertip contour and a horizontal line that was selected to ensure that the area enclosed was equal to *A* (Fig. 7C,D). The position *x*(*t*) of the fingertip was estimated as the *x* coordinate of the centroid of the area enclosed by the curve *C*_*f*_ of every video frame.

Within each frame, the region of interfacial contact between the fingertip and the surface was estimated from the contours of the finger, *C*_*f*_, and the surface, $\hat{h}$. For the purpose of determining the contact interface, a pair of points separated by a distance of 3 pixels (i.e. 70 *μ*m) or less was considered to be in contact.

### Force data processing

The raw signals from the piezoelectric transducers were downsampled and filtered to match the video sampling period of 0.1 ms. The tangential force *F*_*T*_(*t*) low-pass filtered using a zero-phase filter with 500 Hz cutoff frequency. The piezoelectric sensors have a high pass filtering characteristic with approximately 5 Hz cutoff frequency, and the measurements drift slightly during data collection. For each slide, lasting no more than 0.4 s, the drift could be approximated by a linear trend. We removed this artifact by computing the best linear fit to the force signal during the flat segment, and subtracted it from the force measured during the touch interaction. Then the force signals were truncated to the time period corresponding to the finger sliding at a 26 mm window centered at the middle of the surface matching the location of the relief feature. We sampled spatial positions with a resolution of 0.01 mm. The estimates of fingertip position *x*(*t*) were used to infer spatial patterns of the temporal signals, using the same procedure employed in our prior work^20,55^, associating the force *F*_*T*_ to the position *x* at the corresponding time *t*.

### Estimating model parameters

We computed the model forces by computing the integrals in Equation (4) using the contact region ${{\mathscr{C}}}_{i}$ extracted from each trial. Per-subject coefficients of friction *μ* were estimated from the data for each subject via grid search, yielding *μ* = 0.55 and 0.6 for subjects 1 and 2 respectively. We filtered the force estimates using a median filter to remove quantization artifacts that were due to pixelation and illumination errors in the tracked finger contours.

The parameters *p*_0_, *p*_1_ of the force model were estimated via a simplex direct search method^56^, which minimized the normalized mean square error $\varepsilon ({F}_{T},{\hat{F}}_{T})$ (NMSE) between the measured force *F*_*T*_ and the model estimate ${\hat{F}}_{T}$, where

5 $$\varepsilon ({F}_{T},{\hat{F}}_{T})=\frac{\parallel {\hat{F}}_{T}-{F}_{T}\parallel }{\parallel {\hat{F}}_{T}-\overline{{\hat{F}}_{T}}\parallel },\,\overline{{\hat{F}}_{T}}=\frac{1}{L}\,\sum _{x=1}^{L}\,{\hat{F}}_{T}(x)$$

The parameters could vary from trial-to-trial in order to accommodate variations in contact force. To compare the observations and model predictions in different conditions, we computed Pearson’s correlation coefficient values, *ρ*, for the ensemble of measurements in each condition, and the *p*-values for testing the hypothesis that there is no relationship between the observed and measured forces. All correlations were computed on detrended data.

### Data availability

The primary data may be retrieved from the following internet address: https://s3.amazonaws.com/rtlab/MJ-MW-YV-2017-Data.zip.

## Electronic supplementary material

Supplementary Information
