# Supplementary material for: Contact geometry and mechanics predict friction forces during tactile surface exploration
Source: Sci Rep. 2018 Mar 20;8:4868. doi: 10.1038/s41598-018-23150-7 (PMC5861050; doi:10.1038/s41598-018-23150-7)
Supplement: Supplementary file 1 — Supplementary Information [file 41598_2018_23150_MOESM1_ESM.pdf]

Contact geometry and mechanics predict friction forces during tactile surface exploration

Marco Janko, Michael Wiertlewski, Yon Visell

Supplementary Information

Supplementary data 1: Contact lengths

We computed the lengths L_i of each contact region $\mathscr{C}_i$ in each frame, as measured via the fronto-parallel video capture system and resulting video analysis (see Methods), for each finger position during sliding. The resulting patterns are shown in Figure S6, varying little between speeds, with only modest differences between subjects (see Results). The width of the region of contact with the relief feature, and the mean rate of change in the contact length during the ascent and descent of the relief feature were nearly invariant from trial to trial (Figure S6 and Table S1).

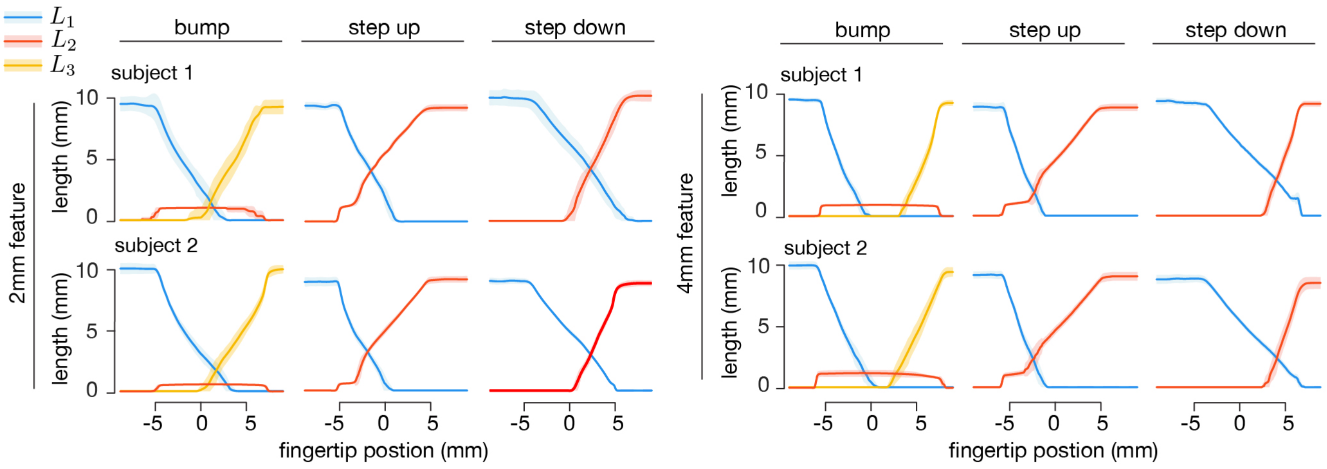

Figure S1. Lengths L_i of finger-surface contact regions, determined from the video capture and analysis, as a function of the fingertip position. Mean of 15 trials L_1 in green, Mean of 15 trials L_2 in red and Mean of 15 trials L_1 in blue. Shaded regions: 1 s.d.

Table S1. Contact length L_i measures; compare with Fig. S6.

	Subject 1						Subject 2					
	2 mm			4 mm			2 mm			4 mm		
	Bump	Step Up	Step Down	Bump	Step Up	Step Down	Bump	Step Up	Step Down	Bump	Step Up	Step Down
Avg. L_1 decrease slope $\frac{dL_1}{dx}$	-1.21	-1.27	-0.92	-1.92	-1.97	-0.77	-1.14	-1.44	-0.83	-1.63	-1.97	-0.79
Avg. L_2 width (mm)	11.0	N/A	N/A	12.9	N/A	N/A	12.0	N/A	N/A	13.7	N/A	N/A
Avg. L_2 (or L_3) increase slope $\frac{dL_2}{dx}$ $(\frac{dL_3}{dx})$	1.36	0.99	1.53	2.21	0.94	2.21	1.15	0.92	1.83	1.60	0.88	2.34

Supplementary data 2: Nonlinear force components

We investigated whether the model predictions would improve if we allowed for a nonlinear polynomial dependence of the deformation component on the surface slope, $dh(x)/dx$. To this end, we replaced $p_1 dh(x)/dx$ in Equation (4) by polynomial functions, with terms $p_n(dh(x)/dx)^n$ of successively higher powers, n up to N, where N is the model order. Fit quality is assessed via the normalized mean square error $\varepsilon(F_T, \hat{F_T})$ (NMSE) between the measured force F_T and the model estimate $\hat{F}_T$, where

$$\varepsilon(F_T, \hat{F_T}) = \frac{\|\hat{F_T} - F_T\|}{\|\hat{F_T} - \overline{\hat{F_T}}\|}, \qquad \overline{\hat{F_T}} = \frac{1}{L}\sum_{x=1}^{L} \hat{F_T}(x) \tag{1}$$

Higher quality fits were achieved with increasing polynomial order, but the best results, as measured by normalized root mean square error ε between the model output and measurements, were nearly achieved by a linear model. The errors for models of quadratic order or higher in $dh(x)/dx$ were negligibly better (Fig. S2). A model of "zeroth" order, which omitted the deformation contribution to sliding friction forces, yielded errors that were 150% higher ($\varepsilon = 1.27$ vs. 0.5).

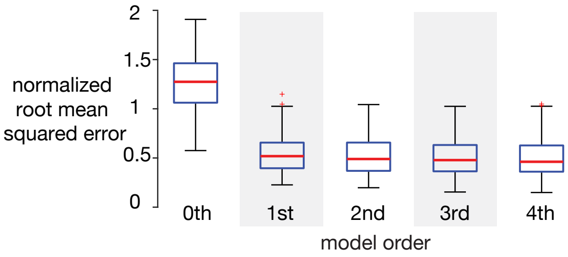

Figure S2. Errors $\varepsilon(F_T, \hat{F_T})$ in the model force predictions vs. polynomial order of σ_p. Each subplot represents 180 values (90 for each subject). The error is dimensionless, owing to the ratio in (1).

Supplementary Information continues on the next page.

Supplementary data 3: Force Model decomposition

The forces as predicted by the model were the sum of two components F_{int} and F_{def} accounting for interfacial shear frictional force and force caused by deformation respectively (Fig. S3).

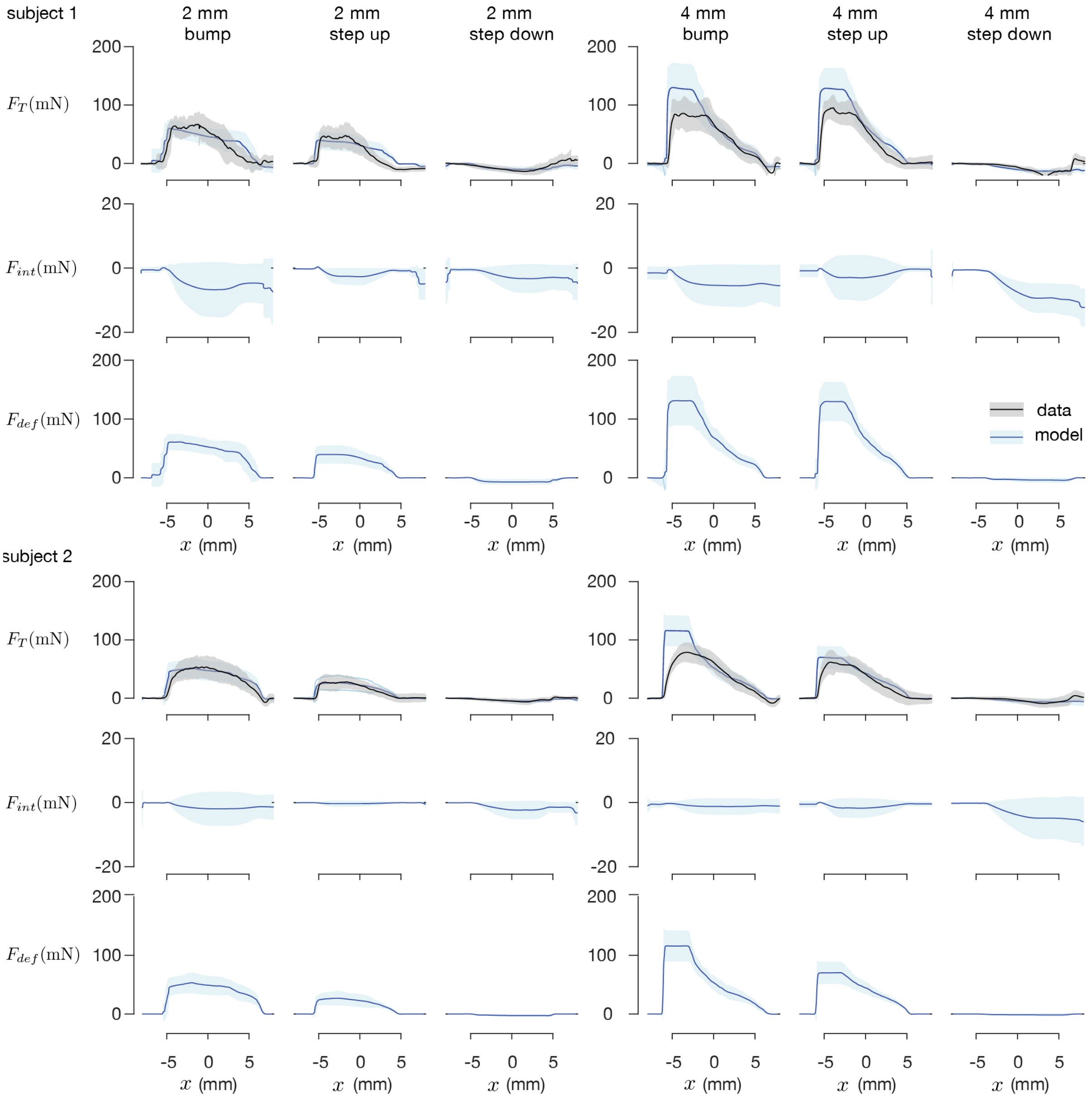

Figure S3. Total force computed by the model and the partial components F_{int} and F_{def}. The force caused by fingertip deformation F_{def} is one order of magnitude larger in the bump and step up surfaces. The shear force estimated F_{int} has comparable level to that of F_{def} only on the step down surfaces. Force estimates $\hat{F}_T$, F_{int}, and F_{def} in blue and force measurements F_T in black. Mean in solid lines, 1 standard deviation in shaded colors.

Supplementary data 4: Model parameter distributions

The force components F_{int} and F_{def} were associated with two parameters p_0, a pressure term, and p_1, which weighted the importance of deformation. Their values were estimated from the data in each trial, accounting for trial to trial variations in contact pressure and force (Fig. S4). The model also depended on a friction coefficient, μ, which was estimated once for each participant, yielding $\mu = 0.55$ for subject 1, and $\mu = 0.6$ for subject 2. By cleaning the surfaces and applying talc, we controlled friction forces during the experiment.

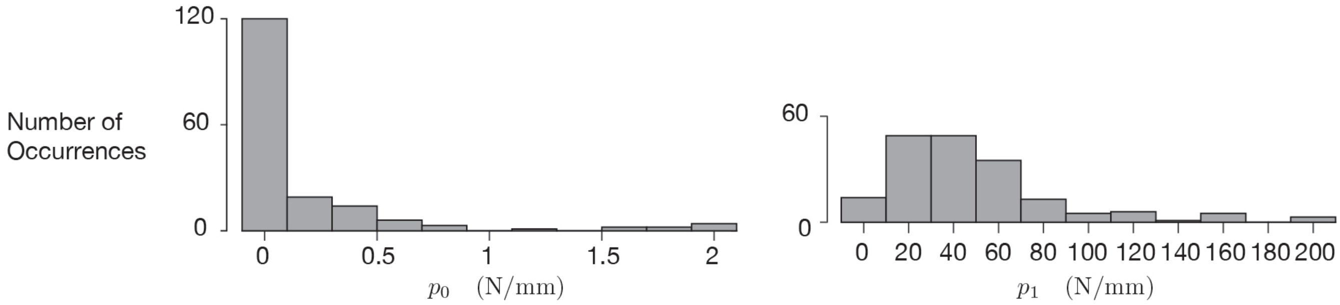

Figure S4. Histograms showing the distribution of the estimated force component parameters p_0 and p_1 for all surfaces, conditions and subjects. **A.** Histogram of the estimated parameters p_0. **B.** Histogram of the estimated parameters p_1.

Supplementary data 5: Fingertip average sliding speed

Subjects were instructed to slide their fingertips on the surfaces with specified sliding speed (40 mm/s, 80 mm/s, or 120 mm/s). The speed varied due to motor behavior and contact with relief features on the surface. We assessed the speeds by measuring the time, t_s, that it took the fingertip to slide across the middle 24 mm of the surface during each trial, and computed the average sliding speed as $v = 24/t_s$. The results (Fig. S5) show show that the average speeds varied around the specified speed, but preserved a general rank ordering.

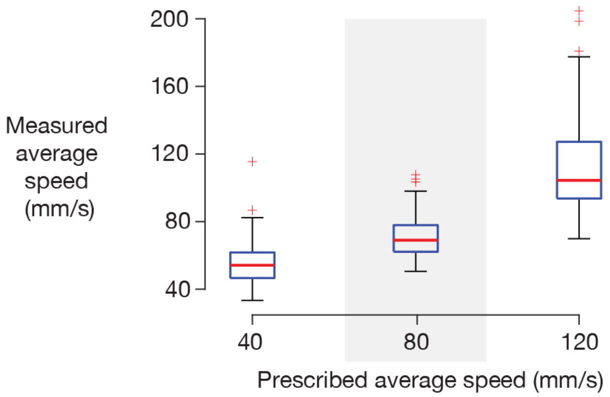

Figure S5. Average sliding speed measurements vs. prescribed average sliding speed. Each boxplot represents 60 values measured under each of the three conditions.

Supplementary data 6: Forces at different speeds, superimposed

Superimposing forces captured at different sliding speeds on each surface reveals that if there is an effect of speed on forces, it is small and inconsistent (Figure S6; same data as shown in Figure 4).

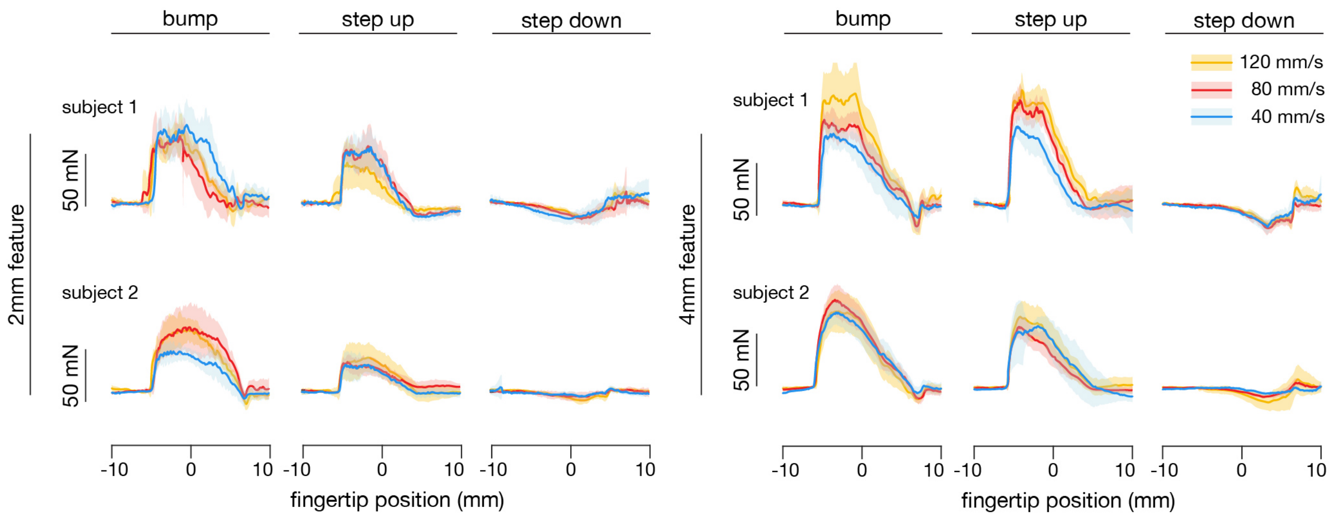

Figure S6. Measured forces $F_T(x)$ grouped by surface and subject (15 trials per case). Mean of 5 trials at 40 mm/s in red, mean of 5 slides at 80 mm/s in blue and mean of 5 trials at 120 mm/s in orange. Shaded regions: 1 standard deviation.

Supplementary data 7: Analytical form of the surface shapes

The surfaces were fabricated using electrical discharge machining, yielding a smooth finish with specified geometry. The height $h(x)$ varied along the length of the surface according to the following expression.

$$h(x) = \begin{cases} 0.2W\sin(\frac{2\pi}{W}x) & \begin{cases} |x| < \frac{W}{2} & \text{Bump} \\ \frac{-W}{2} < x < 0 & \text{Step up} \\ 0 < x < \frac{W}{2} & \text{Step down} \end{cases} \\ 0.2W & \begin{cases} 0 \geq x & \text{Step up} \\ x \leq 0 & \text{Step down} \end{cases} \\ 0 & \text{otherwise} \end{cases} \tag{2}$$
